# Effect of Muscle Energy Technique on Hamstring Flexibility: Systematic Review and Meta-Analysis

**DOI:** 10.3390/healthcare11081089

**Published:** 2023-04-11

**Authors:** Yeh-Hyun Kang, Won-Bae Ha, Ji-Hye Geum, Hyeonjun Woo, Yun-Hee Han, Shin-Hyeok Park, Jung-Han Lee

**Affiliations:** 1 Chuna Manual Medicine Research Group, College of Korean Medicine, Won-Kwang University, 460 Iksan-daero, Iksan 54538, Republic of Korea; 2 Department of Korean Medicine Rehabilitation, College of Korean Medicine, Won-Kwang University, 895 Muwang-ro, Iksan 54538, Republic of Korea; 3 Department of Acupuncture and Moxibustion Medicine, College of Korean Medicine, Won-Kwang University, 895 Muwang-ro, Iksan 54538, Republic of Korea; 4 Research Center of Traditional Korean Medicine, College of Korean Medicine, Won-Kwang University, 895 Muwang-ro, Iksan 54538, Republic of Korea

**Keywords:** hamstring muscles, musculoskeletal manipulations, systematic review, meta-analysis

## Abstract

Since 2005, there have been no systematic reviews on the effects of multiple manual therapies, including muscle energy technique (MET), on the hamstrings. Therefore, this systematic review aimed to provide clinical evidence for the effectiveness of the MET on hamstring flexibility. We queried 10 electronic databases (PubMed, EMBASE, The Cochrane Library, KISS, NDSL, KMBASE, KISTI, RISS, Dbpia, and OASIS) up to the end of March 2022. This study only included randomized controlled trials (RCTs) investigating the use of MET for the hamstring. The literature was organized using Endnote. Literature screening and data extraction were conducted by two researchers independently. The methodological quality of the included RCTs was evaluated using the Cochrane risk-of-bias tool 1.0, and the meta-analysis was performed using RevMan 5.4. In total, 949 patients from 19 RCTs were selected according to the inclusion criteria. During active knee extension tests, the efficacy between MET and other manipulations did not significantly differ. For sit and reach tests, MET groups had higher flexibility compared to stretching (MD = 1.69, 95% CI: 0.66 to 2.73, *p* = 0.001) and no treatment (MD = 2.02, 95% CI: 0.70 to 3.33, *p* = 0.003) groups. No significant differences were observed in the occurrence of adverse reactions. Overall, we found that MET is more efficacious for improving hamstring flexibility compared to stretching and having no treatment during sit and reach tests because it combines isometric contraction with stretching. Owing to clinical heterogeneity, uncertain risk of bias, and the small number of included studies, further high-quality studies should assess the effectiveness of MET.

## 1. Introduction

Anatomically, the hamstring is composed of two parts: the long head and the short head. The long head extends from the ischial tuberosity to the fibula head and acts for hip extension, while the short head extends from the linea aspera of the femur to the fibula head and is involved in knee flexion [1,2]. Consequently, the hamstring has a biomechanical function in the complex movement of the hip, pelvic joint, and spine. Hamstring dysfunction may occur because of a sedentary lifestyle and surgical interventions. During prolonged sitting, hamstring activity may decrease owing to increased pressure on the back of the thigh [3,4]. Furthermore, pelvic posterior tilt and knee flexion can shorten the length of the hamstrings, resulting in tender points and muscle tension in the hamstrings [5,6]. In addition, inflexible and shortened hamstrings may cause low back pain, bad posture, and walking abnormalities [6,7,8,9].

Through muscle flexibility, the body can prevent unnecessary energy expenditure during activity and increase the accuracy of movements, strength activity, and coordination to ensure the range of motion of muscles and joints [10]. In general, cold/thermal therapy, massage, electrotherapy, stretching, neurodynamic treatment, myofascial release, and proprioceptive neuromuscular facilitation (PNF) or muscle energy technique (MET) have been applied to enhance muscle flexibility [11,12,13]. MET has, however, become a more dominant method for reducing musculoskeletal pain and increasing the range of motion of joints in recent years [14].

The MET is one of the manual therapies using voluntary isometric contractions in a target muscle group and is mainly used in osteopathic medicine [15]. MET is similar to proprioceptive neuromuscular facilitation and is known to be more effective in improving the elongation of shortened muscles than conventional static stretching or joint mobilization techniques (by using isometric contractions [16]). Furthermore, it also strengthens muscles, aids in the drainage of body fluids and blood through the lymph or venous pumps, and is effective in increasing the range of motion in joints with limited range of motion [17].

Studies have been conducted on the effectiveness of MET in various diseases such as cervicalgia [18,19,20], chronic low back pain [21], chronic obstructive pulmonary disease (COPD) [22], and symptomatic and asymptomatic subjects [23]; however, studies on the hamstring have not been reported. Furthermore, studies of static stretching [24], Nordic hamstring exercise [25,26,27], eccentric training [28,29], and strength training [30] have been reported on the effects of hamstring strength and flexibility, but no comparative studies with MET have been published. In 2005, a systematic review on the relationship between stretching and hamstrings was published, but systematic reviews on the effects of multiple manual therapies, including MET, on the hamstrings have not been reported since then [31].

Therefore, the study aimed to analyze and present the clinical evidence for the effect of MET on hamstring flexibility by reviewing randomized controlled clinical trials (RCTs).

## 2. Materials and Methods

The protocol of this systematic review has been registered in international registries (PROSPERO: CRD 42022339235). No amendments were issued after the protocol’s registration. This systematic review was conducted in accordance with the Preferred Reporting Items for Systematic Reviews and Meta-Analysis statements [32].

### 2.1. Database Selection and Search

Studies using MET on hamstrings published up to March 2022 were searched in 10 online databases: PubMed (www.pubmed.com), EMBASE (www.embase.com), Cochrane Library (www.thecochranelibrary.com), KISS (kiss.kstudy.com), NDSL (www.ndsl.kr), KMBASE (kmbase.medric.or.kr), KISTI (www.kisti.re.kr), RISS (www.riss.kr), DBpia (www.dbpia.co.kr), and OASIS (oasis.kiom.re.kr).

Search terms ‘hamstring muscle’, ‘biceps femoris’, ‘semimembranosus’, ‘semitendinous’, ‘muscle stretching exercises’, ‘muscle energy technique’, ‘postisometric relaxation’, ‘isometric stretching’, ‘muscle isometric contraction’, and ‘isometrics’ were combined to produce a search expression suitable for each of the following online English databases: PubMed, EMBASE, and Cochrane Library. Search terms ‘muscle energy technique’, ‘MET’, and ‘hamstring’ were combined to produce a search expression suitable for each of the following online Korean databases: KISS, NDSL, KISTI, RISS, Dbpia, and OASIS.

### 2.2. Criteria for Inclusion and Exclusion

In this study, the PICOs (patients, intervention, comparison, outcomes, and study design) model for establishing the search strategy was set as follows. (1) Patients or population: No restrictions on the subjects’ age and sex. Only human clinical trials were included. (2) Intervention: MET applied to the hamstrings. (3) Comparison: No restrictions were placed on the comparator conditions. That is, no treatment, stretching, or other manipulations were allowed; (4) Outcome: The primary outcomes of this review were the active knee extension (AKE), the sit and reach test (SRT), or the straight leg raise test (SLRT). Other measures of pain, including the visual analog scale (VAS), were considered secondary outcomes. (5) Study design: RCTs were included, and pilot studies and preclinical experimental studies were excluded. Only studies written in English and Korean were included in this review.

### 2.3. Literature Selection and Analysis

#### 2.3.1. Literature Search and Selection

Two researchers (K.Y.H., H.W.B.) independently searched and selected literature, and in case of disagreement, a discussion was conducted between them. Initially, the title and abstract of the searched literature were reviewed, followed by the research design, target patient, intervention, control group, evaluation index, and results. A detailed review of the selected literature was conducted, and finally, a selection of studies that met the selection criteria was made.

#### 2.3.2. Literature Search and Selection

For literature analysis, two researchers (K.Y.H., H.W.B.) reviewed the full text of the selected literature, extracted information including the study design, and summarized the intervention, control group, evaluation index, and main results in a table. In case of disagreement between the two independent researchers when extracting data, the opinion of a third researcher (L.J.H.) was considered.

Using Cochrane’s Risk of Bias (ROB) evaluation table, two independent researchers (K.Y.H., H.W.B.) assessed 7 items of literature selected to assess the risk of bias of the investigated RCTs. If there was a disagreement between the evaluators, it was reconsidered and agreed upon through discussion with the third researcher (L.J.H.).

### 2.4. Data Extract

Data such as author, year, intervention, control group, number of subjects, evaluation index, and results were extracted from each paper by reviewing the original text of the finally selected literature. In the meta-analysis, Cochrane’s Review Manager (RevMan) Ver. 5.4 (Copenhagen, Denmark) was used. Continuous results were analyzed through mean difference (MD) and 95% confidence interval (CI). Higgin I^2^ values of <50% and ≥50% were interpreted as low and heterogeneity, respectively.

## 3. Results

### 3.1. Literature Selection

**Figure 1:**
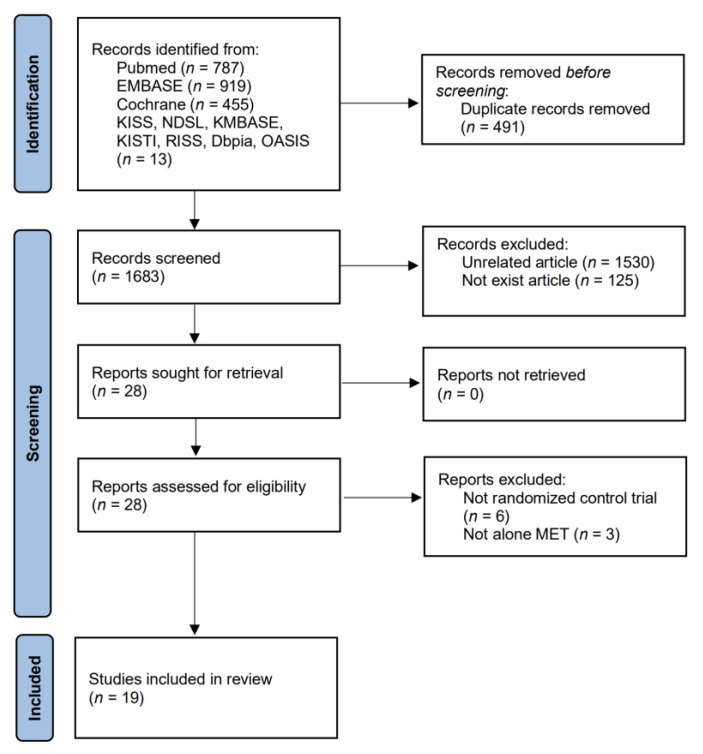
A flow chart diagram describing the trial selection process. *n*: number of articles; MET: muscle energy technique; RCT: randomized controlled trial.

A total of 2174 documents published up to March 2022 were retrieved through the search of 10 online databases. In addition, 491 duplicate documents were excluded. After screening the titles and abstracts, 1655 articles were excluded due to irrelevancy or the unavailability of the original text (since the authors could not be contacted). Finally, 19 articles were selected after excluding 6 articles that were not RCTs and 3 articles in which the effect of MET alone was unknown (Figure 1).

### 3.2. Literature Analysis

#### 3.2.1. Study Overview

In total, 19 RCTs were finally selected which evaluated 949 cases of hamstrings. In all 19 trials, MET was used as an intervention method in the test group. In the control group, the active release technique (ART), Mulligan technique, neural tissue mobilization, neural slump stretch, whole body vibration (WBV), and other manual therapies, such as physical therapy, were used as an intervention. In addition, passive and static stretching or different types of MET were used as an intervention (Table 1).

#### 3.2.2. Evaluation Index

In the selected 19 randomized controlled trials, 15 evaluation indices were used. As an evaluation index, 11 studies used the AKE, seven used the SRT, and four used the VAS. Furthermore, as evaluation indices, SLRT, the Oswestry disability index (ODI), the Korean version of Oswestry disability index (K-ODI), the passive knee extension test (PKET), and the fear avoidance beliefs questionnaire (FABQ) were all used in two studies. The Oxford knee score (OKS), stiffness, pressure pain (PP), the fingertip-to-floor test (FTF), the modified–modified Schober test (MMS), hip flexion range of motion (HFROM), and pelvic inclination (PI) were used as an evaluation index in one study each.

As for the evaluation index, the AKE is a test method that measures the level of tension in the hamstring muscles by actively extending the knee while the hip joint is bent at 90°. The existing SLR test is more useful as a neurodynamic test rather than a muscle length assessment because it is difficult to determine the exact cause of muscular or other neurological problems; therefore, the use of the AKE test is increasing [49]. The SRT is a test in which the hand is stretched forward in a sitting position, and the subject must completely touch the sole of the leg to be measured with the measuring device and avoid bending the knee. The non-measured leg is bent at 135° at the hip joint and 90° at the knee joint so that the sole touches the floor. Subjects are instructed to push the measuring plate by bending the hip joint and torso as far forward as possible using the arms, elbows, fingers, and palms fully extended [50,51].

The ODI is a general examination for spinal diseases and consists of 10 items that evaluate the level of pain and limitation of daily life with respect to physical activity [52]. The FTF is used to measure the degree of overall bending of the torso. The test’s motions are as follows: Stand upright in front of a 20 cm high box, place your toes on the end of the box, and bend your torso forward; the distance between the subject’s fingertips and the top of the chair is measured using a tape measure [53]. The FABQ is used to evaluate psychosocial characteristics. The MMS is used to measure the amount of motion in the waist during trunk bending, and the HFROM is used to evaluate the degree of hip joint bending during trunk bending.

### 3.3. Therapeutic Effect

#### 3.3.1. Comparison of MET Group and Stretching Group

Intergroup analysis was performed in six of eight studies comparing MET and stretching. Kang [47] and Lim [10] reported that the MET group was more effective on the SRT index, while Park [45] reported that the MET group was more effective on K-ODI, VAS, FABQ, and PP indices. In contrast, Banerjee [42] reported that the stretching group was more effective on the AKE index, and Cha reported that the stretching group was more effective on the FTF index. Payla [33] and Sathe [34] analyzed the AKE and SRT indices, and there was no statistically significant difference between the two groups.

**Figure 2:**
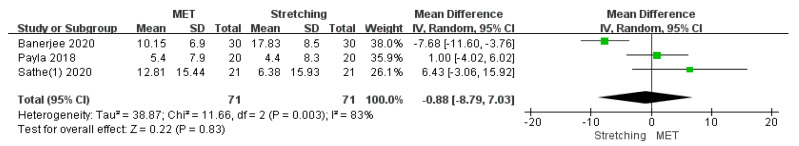
Meta-analysis of active knee extension (AKE) outcomes compared of AKE between muscle energy technique (MET) and stretching groups [33,34,42]. SD: standard deviation; CI: confidence interval.

In a meta-analysis of three studies [33,34,42] using AKE as an evaluation index immediately after treatment, the results showed a mean difference (MD) of −0.88 ([95% CI –8.79, 7.03], *p* = 0.83, I^2^ = 83%), indicating that it was not possible to determine which intervention had a more statistically significant therapeutic effect between the MET and stretching groups. In addition, a high level of heterogeneity between studies was confirmed (Figure 2).

**Figure 3:**
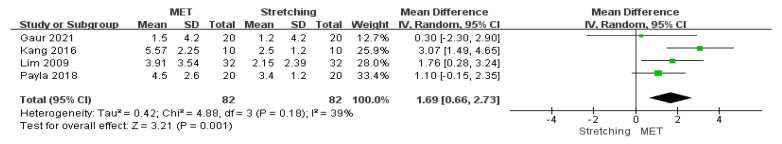
Meta-analysis of sit and reach test (SRT) outcomes of SRT compared between muscle energy technique (MET) and stretching groups [10,33,43,47]. SD: standard deviation; CI: confidence interval.

In a meta-analysis of four studies [10,33,43,47] using SRT as an evaluation index immediately after treatment, the results showed an MD of 1.69 ([95% CI 0.66, 2.73] *p* = 0.001, I^2^ = 39%), indicating that the MET group showed a statistically significant treatment effect compared to that of the stretching group. In addition, there was a moderate level of heterogeneity between studies (Figure 3).

#### 3.3.2. Comparison between MET Group and ART Group

According to the intergroup analysis conducted among the three studies comparing the MET and the ART technique, Khan [41] reported that the ART group was more effective on the AKE index. Furthermore, Gaur [43] analyzed the AKE and SRT indices, and Amin [36] analyzed the SRT index; however, there was no statistically significant difference between the two groups.

**Figure 4:**
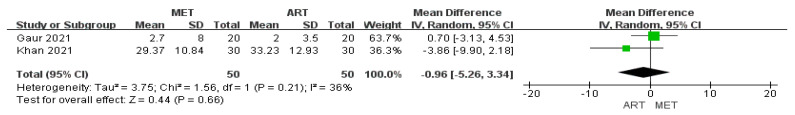
Meta-analysis of active knee extension (AKE) outcomes compared between muscle energy technique (MET) and active release technique (ART) groups [41,43]. SD: standard deviation; CI: confidence interval.

In a meta-analysis of two studies [41,43] using AKE as an evaluation index immediately after treatment, the results showed an MD of −0.96 ([95% CI –5.27, 3.34] *p* = 0.66, I^2^ = 36%), indicating that it was not possible to determine which intervention had a more statistically significant therapeutic effect between the MET and ART groups (Figure 4).

#### 3.3.3. Comparison of the MET Group and the Mulligan Group

According to an intergroup analysis conducted among the two studies comparing the MET and the Mulligan technique, Amin [36] reported that the MET group was more effective on the AKE index, but did not report a statistically significant difference between the two groups for the SRT index. Tariq [39] reported that the Mulligan group was more effective on the VAS index, and there was no statistically significant difference between the two groups for the AKE and KOS indices.

**Figure 5:**
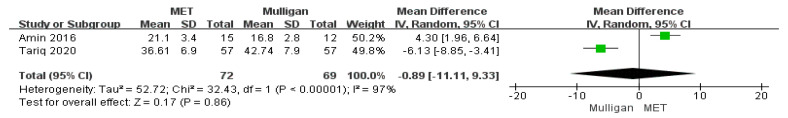
Meta-analysis of active knee extension (AKE) outcomes compared between muscle energy technique (MET) and the Mulligan technique groups [36,39]. SD: standard deviation; CI: confidence interval.

In a meta-analysis of two studies [36,41] using AKE as an evaluation index after four weeks of treatment three times a week, the results showed an MD of −0.89 ([95% CI −11.11, 9.33] *p* = 0.86, I^2^ = 97%), indicating that it was not possible to determine which intervention had a more statistically significant therapeutic effect between the MET and Mulligan groups. In addition, a high level of heterogeneity between studies was confirmed (Figure 5).

#### 3.3.4. Comparison of MET Group and Non-Treatment Group

Intergroup analysis was performed in three of five studies comparing muscle energy techniques and stretching. Lim [10] reported that the MET group was more effective on the SRT index, Ballantyne [17] on the PKET index, and Cha [46] on K-ODI, VAS, FABQ, and FTF indices.

**Figure 6:**
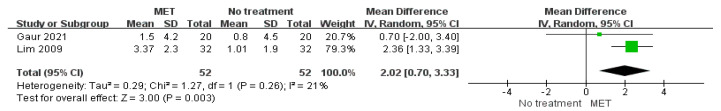
Meta-analysis of sit and reach test (SRT) outcomes compared between muscle energy technique (MET) and no-treatment groups [10,43]. SD: standard deviation; CI: confidence interval.

In a meta-analysis of two studies [10,43] using SRT as an evaluation index immediately after treatment, the results showed an MD of 2.02 ([95% CI 0.70, 3.33] *p* = 0.003, I^2^ = 21%), indicating that the MET group was more statistically significant than the untreated group. In addition, a low level of heterogeneity between studies was confirmed (Figure 6).

#### 3.3.5. Comparison between MET Groups

According to the intergroup analysis conducted among three of four studies comparing MET, Hwang [48] reported that the MET-ATM group was more effective than the MET group on AKE and SRT indices. Sathe [35] analyzed the AKE index between the PIR-MET and RI-MET groups, and Shahzad [38] analyzed the SLRT and ODI indices between the concentric and eccentric MET groups. However, there was no statistically significant difference between groups.

#### 3.3.6. Comparison of MET Group and Other Treatment Groups

According to an intergroup analysis conducted among three studies comparing MET and other treatment groups (mobilization, vibration, and physical therapy), Park [45] reported that the MET group was more effective on VAS, ODI, FABQ, and PP indices. In addition, Azizi [44] analyzed AKE, SRT, and stiffness indices, while Patel [37] analyzed VAS, ODI, and SLRT indices. However, there was no statistically significant difference between the groups.

#### 3.3.7. Adverse Reaction

There were no adverse reactions reported in all 19 studies.

#### 3.3.8. Assessment of the Risk of Bias

Using Cochrane’s RoB tool to assess the risk of bias in the 19 selected articles, 10 out of 19 articles in randomized order were rated as low risk owing to the use of an online random tool, random number table, and lottery. Among the remaining articles, one was evaluated as high risk because of using quasi-random sampling, and eight were evaluated as an uncertain risk because only randomization was mentioned.

In concealment of assignment order among selection bias, 3 out of 19 articles were rated as low risk because of independent central randomization and management. The remaining 16 articles were randomly assigned but were evaluated as an uncertain risk because serial numbers, sealing, and transparency was not described.

**Figure 7:**
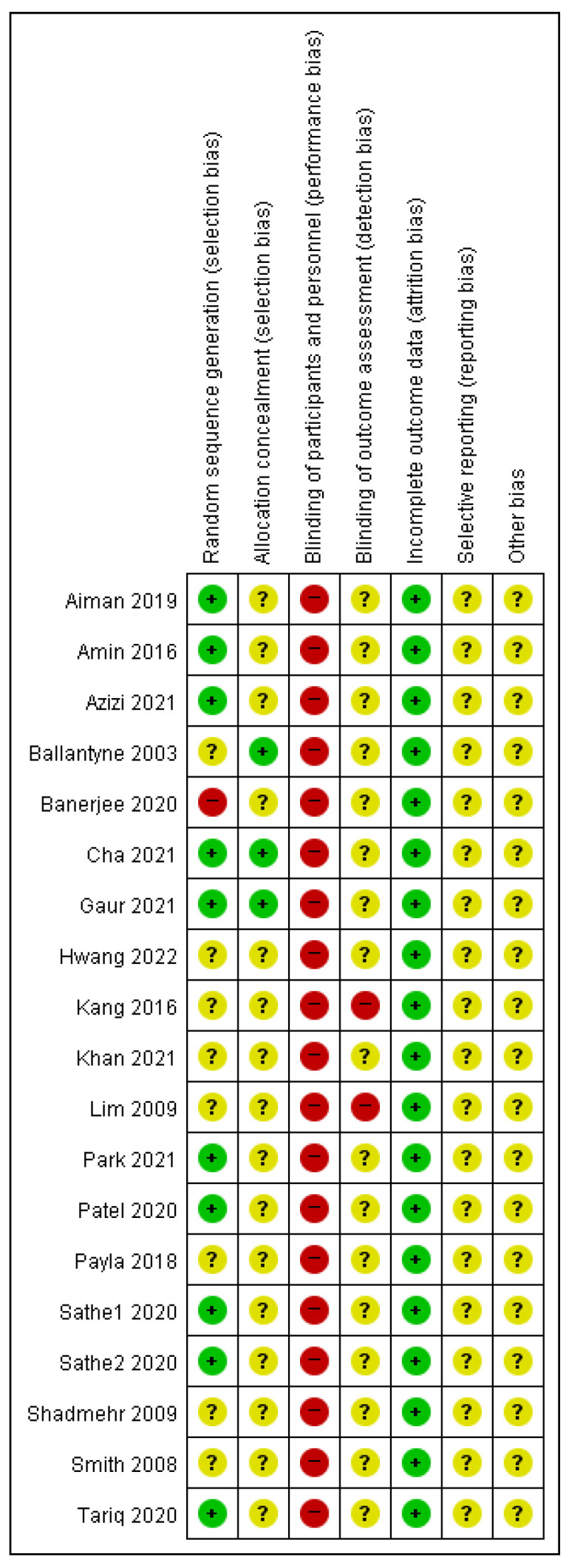
Risk of bias summary [10,15,17,33,34,35,36,37,38,39,40,41,42,43,44,45,46,47,48]. (+) = low risk of bias; (?) = unclear risk of bias; (−) = high risk of bias.

Owing to the nature of the MET, blinding the operator and patient was difficult; therefore, the risk of performance bias was evaluated as high in all 19 articles. When evaluating the outcome of the intervention, 2 out of 19 articles were evaluated as high risk of detection bias owing to the fact that the researcher and the outcome evaluator measured them in the same room. In the attrition bias, all 19 articles were evaluated as low risk because there was little or no information about missing values, and in the reporting bias, all 19 articles were rated as uncertain risk because there was not sufficient information for judgment (Figure 7).

## 4. Discussion

### 4.1. Summary of Findings

In this study, we conducted a meta-analysis to determine whether the MET clinically enhances hamstring flexibility. Among 19 previous studies, 17 conducted statistical comparisons between the MET and control groups. As a result, the MET group rendered higher treatment effects over VAS [39], PKET [17], K-ODI, FABQ, FTF, PP [45], and SRT [10,47] indicators than control groups (*p* < 0.05). The stretching and ART groups were more effective according to AKE indicators than the MET groups (*p* < 0.05) [17,41]. No adverse reaction reported.

Meta-analyses were conducted for 9 of the 19 studies, classifying them by the method of applying intervention, control group, and evaluation indicators. As a result, we coherently found that the MET showed significant effects on SRT increase compared to the stretching and non-treatment groups. However, in analyses on AKE, no effect difference was found among the MET, stretching, ART, and Mulligan groups; the reason appears to be that AKE is more subject to errors by observer and observation method, unlike SRT, based on fixed posture.

### 4.2. Clinical Implication

The MET was designed by phrenologists including T.J. Ruddy and Fred Mitchell Sr. to improve musculoskeletal function and relieve pain, and more recently developed by researchers including Karel Lewit and Bladimir Janda. This technique aims to cure the symptoms by extending and easing highly tense muscles through precise control and integration of isometric, isotonic contraction accompanied by the patient’s motion [54]. Generally, the MET can be used to strengthen the muscles and operate joints in contraction, contracture, spasticity, or weakness. Furthermore, it also serves for operating joints [55]. While the physiological mechanism behind MET treatment effects has not yet been clarified, it is hypothesized that they are associated with various neurologic and biomechanical factors including hypoalgesia, proprioception, motor programming and control, and changes in tissue fluids [56].

While all 19 studies applied the MET for intervention groups, the intervention methods were slightly different across those studies. Stretching was set as a control group in 8 of 19 articles. During stretching, the hip joint was flexed on the exercise side with the knee joint extended, and the posture was maintained within a range where the pain was not felt along with repeated rests. In addition, three studies applied the ART—a musculoskeletal manual treatment—to the control group. Two others used the Mulligan technique; five applied no treatment at all to the control group. Lastly, another three studies applied neural tissue mobilization, WBV, and physical therapy for control groups, and four studies made comparisons between distinct MET groups. 

In the literature, the ART is a treatment method for cumulative injury disorder. Accordingly, in conjunction with stretching, pressure is applied to change the pose from shortening to lengthening after longitudinal contact along the direction of the fibrous tubercle of contracted tissues [57]. As such, the adhesion among soft tissues can be relieved. The Mulligan technique is a joint mobilization method developed by Brian R. In addition, the Mulligan technique can increase the angle of the lower extremity in patients with limited back pain or radiation through bent leg lift, two-leg rotation, and traction [58]. Neural mobilization is a treatment method of separating nerves from surrounding tissues by inducing nerve movement when the nerves are caught, compressed, and fail to function properly [59,60,61]. The WBV is a relatively safe and convenient technique, in which vibration is transmitted through the whole body or to a specific area through a platform; it is a relatively new technique and has recently become a popular option for rehabilitation and sports [62]. Physical therapy used in this review as an intervention consisted of 20 min of warm compresses and proximal infrared rays, and 15 min of interference wave current therapy.

No adverse events were observed in all 19 studies. This indicates that the MET and other manipulations, including stretching and mobilization, are non-invasive and safe treatments. In particular, the MET employs gradual power and proper speed during technique, unlike the high-velocity low-amplitude (HVLA) techniques with rapid use of force. 

### 4.3. Limitations

Admittedly, there are certain limitations in our study. First, we could not make a more direct comparison among different literature results because the MET treatment methods and frequencies were not coherent among them. Second, no literature provides information regarding contortion risk assessment indicators when blindly assessed risk is high or uncertain. As a result, we could not address this limitation. Third, we included only English and Korean databases in this review. Thus, further research is recommended including other languages, such as Hindi and Mandarin. Finally, most studies relied on short-period observation for a small sample, and mainly for healthy adults <45 years old; therefore, further studies are needed on larger sample sizes with a higher age group, and statistical analysis about sensitivity and publish bias would be possible.

In the bias risk assessment, only 10 out of 19 studies specified that their randomization was made using the order or randomizing tool, random number tables, and drawing lots; however, no other study mentioned the randomization method. Furthermore, only three studies made sure that the randomization order was hidden (not disclosed) to participants; therefore, we cannot be sure that the selection bias was certainly prevented. That is, there are concerns about internal validity issues. Owing to the nature of MET, bias risk would also be high because none of the 19 studies conducted blind examinations for researchers and participants. Therefore, subsequent studies must specify the randomization order and its methods.

### 4.4. Suggestions for Further Research

Despite these limitations, the present study’s significance lies in the fact that it successfully addressed the grounds for the MET’s clinical effects on muscle flexibility based on a thorough search of both domestic and international databases. Furthermore, we confirmed its relative effectiveness over treatment by combining stretching and mobilization, especially in RI or PIR where muscle contractions are used. Although there were certain difficulties in the control group setting due to the nature of the muscle energy technique, we cannot ignore the endeavors made by various researchers [63,64] to overcome them. We would also like to emphasize the need for further randomized studies with a larger sample and coherence in treatment frequency and procedures, where post-blind assessment is allowed.

## 5. Conclusions

In conclusion, this systematic review and meta-analysis demonstrated that the use of MET is more efficacious for hamstring flexibility than stretching or having no treatment, based on the SRT index. On the other hand, MET and other manipulations do not statistically differ in efficacy based on the AKE index. It is thought that the reason for these results is that MET combines isometric contraction with stretching, unlike other techniques. Although MET is accompanied by such muscle contraction, since no adverse effects were reported, the clinical use of MET can be recommended to increase the flexibility of the hamstring muscle. 

## Figures and Tables

**Table 1:** Clinical studies on the use of MET on the hamstrings.

First Author	Age	Method	Treatment	Outcomes	Results
Payla (2018) [33]	18–25	A: MET (n = 20) B: Passive stretching (n = 20)	One time	(1) AKE (2) Sit and reach test	1. Mean difference (Post–Pre) (1) Shortly after treatment; A: 5.4°, B: 4.4° After 1 h; A: 3.9°, B: 2.9° (2) Shortly after treatment; A: 4.5 cm, B: 3.4 cm After 1 h; A: 3.3 cm, B: 2.5 cm 2. Comparison between groups Shortly after treatment; A > B (*p* = 0.11) After 1 h; A > B (*p* = 0.11) Shortly after treatment; A > B (*p* = 0.10) After 1 h; A > B (*p* = 0.18)
Smith (2008) [15]	18–65	A: MET with 30 s stretch (n = 20) B: MET with 3 s stretch (n = 20)	1 session/week for 2 weeks	(1) AKE	1. Mean difference (Post–Pre) (1) Shortly after treatment; A: 8.5°, B: 7.9° After 1 weak; A: 6.1°, B: 6.4° 2. No comparison between groups
Sathe (1) (2020) [34]	No age restriction	A: MET (n = 21) B: Passive stretching (n = 21)	One time	(1) AKE	1. Mean difference (Post–Pre) (1) Shortly after treatment; A: 12.8°, B: 6.4° 2. Comparison between groups (1) A > B (*p* = 0.44)
Sathe (2) (2020) [35]	25–60	A: MET (PIR) (n = 20) B: MET (RI) (n = 20)	One time	(1) AKE	1. Mean difference (Post–Pre) (1) Shortly after treatment; A: 13.2°, B: 5.5° 2. Comparison between groups (1) A > B (*p* = 0.39)
Amin (2016) [36]	18–26	A: MET (n = 15) B: Active Release Technique (n = 13) C: Mulligan’s technique (n = 12) D: No treatment (n = 17)	3 sessions/week for 1 month	(1) AKE (2) Sit and reach test	1. Mean difference (Post–Pre) (1) After 1 month; A: 21.1°, B: 21.4°, C: 16.8°, D: −0.3° (2) After 1 month; A: −6.3 cm, B: −6.7 cm, C: −5.3 cm, D: −0.9 cm 2. Comparison between groups (1) A > C (*p* < 0.0001), B > C (*p* < 0.0001) (2) No comparison between groups
Patel (2020) [37]	25–40	A: MET (n = 10) B: Neural tissue mobilization (n = 10)	5 days per weeks for 2 weeks	(1) VAS (2) ODI (3) SLRT	1. Mean difference (Post–Pre) (1) After 2 weeks; A: −2.4, B: −1.9 (2) After 2 weeks; A: −11.8, B: −9.8 (3) After 2 weeks; A: 4.6°, B: 4.5° 2. Comparison between groups (1) A > B (*p* = 0.6691) (2) A > B (*p* = 0.3957) (3) A > B (*p* = 0.6289)
Shahzad (2019) [38]	20–35	A: Concentric MET (n = 30) B: Eccentric MET (n = 30)	3 sessions/week for 4 weeks	(1) right SLRT (2) left SLRT (3) ODI	1. Mean difference (Post–Pre) (1) After 4 weeks; A: 29.5°, B: 29° (2) After 4 weeks; A: 28.5°, B: 23.5° (3) After 4 weeks; A: −4, B: −10 2. Comparison between groups (1) A > B (*p* = 0.730) (2) A > B (*p* = 0.84) (3) A < B (*p* = 0.122)
Tariq (2020) [39]	40–65	A: PIR-MET (n = 57) B: Mulligan’s technique (n = 57)	3 sessions/week for 4 weeks	(1) VAS (2) AKE (3) OKS	1. Mean difference (Post–Pre) (1) After 1 week; A: −1, B: −1.6 After 2 weeks; A: −2, B: −3.5 After 3 weeks; A: −3.1, B: −5.4 After 4 weeks; A: −4.1, B: −6.7 (2) After 1 week; A: −10.1°, B: −12.9° After 2 weeks; A: −20.2°, B: −25.4° After 3 weeks; A: −30.0°, B: −36.6° After 4 weeks; A: −36.6°, B: −42.7° (3) After 1 week; A: 3.1, B: 4.4 After 2 weeks; A: 6.3, B: 9.2 After 3 weeks; A: 9.6, B: 13.7 After 4 weeks; A: 12.9, B: 18.1 2. Comparison between groups (1) A < B (*p* < 0.000) (2) A < B (*p* > 0.358) (3) A < B (*p* > 0.634)
Shadmehr (2009) [40]	20–25	A: MET (n = 15) B: Static stretch (n = 15)	4-week period (3 alternate days per week)	(1) Passive knee extension test	1. Mean difference (Post–Pre) (1) After 4 weeks; A: 22.1°, B: 18.9° 2. No comparison between groups
Khan (2021) [41]	18–35	A: MET (n = 30) B: Active Release Technique (n = 30)	One time	(1) AKE	1. Mean difference (Post–Pre) (1) Shortly after treatment; A: 29.4°, B: 33.2° 2. Comparison between groups (1) A < B (*p* < 0.05)
Banerjee (2020) [42]	18–35	A: MET (n = 30) B: Stretching (n = 30)	One time	(1) AKE	1. Mean difference (Post–Pre) (1) Shortly after treatment; A:10.2°, B: 17.8° 2. Comparison between groups (1) A < B (*p* < 0.05)
Gaur (2021) [43]	20–40	A: MET (n = 20) B: Active Release Technique (n = 20) C: No treatment (n = 20)	One time	(1) AKE (2) Sit and reach test	1. Mean difference (Post–Pre) (1) Shortly after treatment; A:2.7°, B:2.0° After 10 min; A: 4.0°, B: 3.3° (2) Shortly after treatment; A: 3.0 cm, B: 3.0 cm After 10 min; A: 8.2 cm, B: 5.3 cm 2. No comparison between groups
Ballantyne (2003) [17]	18–45	A: MET (n = 20) B: No treatment (n = 20)	One time	(1) Passive knee extension test	1. Mean difference (Post–Pre) (1) torque 1; A: −0.7°, B: −2.7° torque 2; A: 2.7°, B: 0.1° 2. No comparison between groups
Azizi (2021) [44]	18–30	A: PIR-MET (n = 28) B: Whole-body vibration (n = 28)	One time	(1) AKE (2) Modified Sit and reach test (3) Stiffness	1. Mean difference (Post–Pre) (1) Shortly after treatment; A: 11.2°, B: 10.6° (2) Shortly after treatment; A: 2.8 cm, B: 2.4 cm (3) Shortly after treatment; A: 1.8 cm, B: 1.6 cm 2. Comparison between groups (1) A > B (*p* = 0.188) (2) A > B (*p* = 0.147) (3) A > B (*p* = 0.197)
Park (2021) [45]	30–40	A: MET (n = 10) B: Physical therapy (n = 10) C: Stretching (n = 10)	5 sessions/week for 3 weeks	(1) K-ODI (2) VAS (3) FABQ (4) PP	1. Mean difference (Post–Pre) (1) After 3 weeks; A: −8.4, B: −3.4, C: −5.0 (2) After 3 weeks; A: −1.8, B: −1.0, C: −1.0 (3) After 3 weeks; A: −15.0, B: −9.9, C: −4.3 (4) After 3 weeks; A: 5.7, B: 3.1, C: 1.9 2. Comparison between groups (1) A < B,C (*p* < 0.001) (2) A < B (*p* < 0.05) (3) A < B,C (*p* < 0.001) (4) A > B (*p* < 0.05)
Cha (2021) [46]	Twenties–thirties	A: MET (n = 19) B: Stretching exercise (n = 16) C: No treatment (n = 17)	2 sessions/week for 4 weeks	(1) K-ODI (2) VAS (3) FABQ (4) FTF (5) MMS (6) HFROM	1. Mean difference (Post–Pre) (1) After 4 weeks; A: −6.9, B: −7.1, C: −2.9 (2) After 4 weeks; A: −2.5, B: −2.4, C: −1.1 (3) After 4 weeks; A: −15.8, B: −17.2, C: −4.2 (4) After 4 weeks; A: 10.8 cm, B: 11.5 cm, C: −0.7 cm (5) After 4 weeks; A: 0.9 cm, B: 1.2 cm, C: 0.8 cm (6) After 4 weeks; A: −14.6°, B: −9.2°, C: −9.0° 2. No comparison between groups
Kang (2016) [47]	College students	A: PIR-MET (n = 10) B: Stretching (n = 10)	One time	(1) Sit and reach test	1. Mean difference (Post–Pre) (1) Shortly after treatment; A: 7.6 cm, B: 4.4 cm After 10 min; A: 5.6 cm, B: 2.5 cm 2. Comparison between groups (1) Shortly after treatment; A > B (*p* < 0.05) After 10 min; A > B (*p* < 0.05)
Lim (2009) [10]	College students	A: PIR-MET (n = 32) B: Stretching (n = 32) C: No treatment (n = 32)	One time	(1) Sit and reach test	1. Mean difference (Post–Pre) (1) Shortly after treatment; A: 3.4 cm, B: 1.9 cm, C: 1.0 cm After 10 min; A: 3.9 cm, B: 2.2 cm, C: 1.5 cm 2. Comparison between groups (1) Shortly after treatment; A > B,C (*p* < 0.01) After 10 min; No comparison
Hwang (2022) [48]	College students	A: MET (n = 16) B: MET-ATM (n = 16) (Active therapeutic movement)	Once each at weekly intervals (Crossover study)	(1) AKE (2) Sit and reach test (3) Pelvic inclination (forward bending)	1. Mean difference in LBP group (Post–Pre) (1) Shortly after treatment; A: 3.6°, B: 8.0° (2) Shortly after treatment; A: 2.3 cm, B: 4.4 cm (3) Shortly after treatment; A: 3.9°, B: 3.4° 2. Mean difference in non-LBP group (Post–Pre) (1) Shortly after treatment; A: 3.8°, B: 8.4° (2) Shortly after treatment; A: 2.3 cm, B: 4.4 cm (3) Shortly after treatment; A: 3.8°, B: 6.4° 3. Comparison between groups (1) A < B (*p* < 0.01) (2) A < B (*p* < 0.01) (3) No comparison

Notes. AKE: active knee extension test; VAS: visual analog scale; ODI: Oswestry disability index questionnaire; SLRT: straight leg raise test; OKS: Oxford knee score; PP: pressure pain; FTF: fingertip-to-floor test; K-ODI: Korean version of Oswestry disability index; FABQ: fear avoidance beliefs questionnaire; MMS: modified–modified Schober test; HFROM: hip flexion range of motion.

## Data Availability

The data supporting this systematic review and meta-analysis are from previously reported studies and datasets, which have been cited. The processed data are available from the corresponding author upon request.
